# Effects of GLP‐1 Receptor Agonists on Paediatric Population in a Real World Setting

**DOI:** 10.1002/edm2.70053

**Published:** 2025-04-22

**Authors:** A. G. Barajas, Charles A. Gagnon‐Vargas, Jessica A. Schmitt

**Affiliations:** ^1^ University of Alabama at Birmingham Marnix E. Heersink School of Medicine Birmingham Alabama USA; ^2^ Division of Pediatric Endocrinology and Diabetes, Department of Pediatrics University of Alabama at Birmingham Marnix E. Heersink School of Medicine Birmingham Alabama USA

**Keywords:** adolescent, diabetes mellitus, drug utilisation, glucagon‐like peptide‐1 receptor agonists, type 2

## Abstract

**Objective:**

Incidence of type 2 diabetes mellitus (T2DM) and obesity is increasing in children. We aimed to observe the metabolic health effects of glucagon‐like peptide 1 (GLP‐1) receptor agonists in paediatric patients with T2DM in a real‐world setting.

**Methods:**

A retrospective chart review of patients aged 0 to 18 years with T2DM who were started on a GLP‐1 receptor agonist between August 2019 and August 2023 and followed for up to 24 months was included in this study.

**Results:**

321 patients were included in the analysis. After 12 months of treatment with a GLP‐1 receptor agonist, haemoglobin A1c (HbA1c) was reduced by −1.04% ± 2.3% (*p* < 0.001). At 24 months, HbA1c was similar to baseline (8.3% ± 2.5% vs. 7.9% ± 2.5%, *p* = 0.24). There was a significant (*p* < 0.05) decrease in the use of metformin, basal insulin, and bolus insulin at one year which was sustained at the two‐year follow‐up for metformin (*p* = 0.002). Overall, there was no significant change in BMI nor other metabolic parameters while undergoing treatment with a GLP‐1 receptor agonist.

**Conclusion:**

Paediatric patients with T2DM using GLP‐1 receptor agonists experienced a significant decrease in HbA1c after 12 months of use, which was not sustained at 24 months. However, patients had a reduction in insulin and metformin use at 12 months. No significant impact was appreciated on BMI or other metabolic variables.

## Graphical abstract

A retrospective review of 300+ children with type 2 diabetes on GLP‐1 receptor agonists showed minimal weight effect, unsustained A1c improvement, and reduced polypharmacy in real‐world settings.

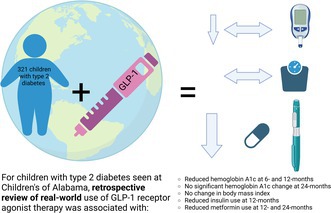

## 1 Introduction

Type 2 Diabetes Mellitus (T2DM) in young people is projected to quadruple by 2050 [1]. A similar trend can be seen in childhood obesity, and the World Health Organisation recently announced 340 million adolescents and 39 million children worldwide are considered obese [2]. These rates are intricately linked as obesity in childhood has been shown to increase the risk of developing T2DM [3]. This relationship is of special concern in paediatric patients, a population that experiences accelerated Beta‐cell dysfunction, insulin resistance, and diabetes‐related complications [4, 5, 6].

Despite the accelerated rates for diabetes‐related complications for youth with T2DM, the number of approved pharmacologic agents has lagged behind that for adults with T2DM. Until 2019, the only FDA‐approved medications for children with T2DM were metformin and insulin [6]. However, treatment with insulin can lead to weight gain. In addition, studies have shown high therapeutic failure rates with metformin in those with rapid beta‐cell deterioration, with no significant effects on weight [7]. GLP‐1 receptor agonists address this concern in paediatric patients, as this class of medications promotes endogenous release of insulin, promotes pancreatic beta‐cell proliferation, and reduces beta‐cell apoptosis [4, 8]. Currently, three GLP‐1 receptor agonists—semaglutide, dulaglutide, and liraglutide—are approved for T2DM in youth. Of these, only semaglutide and liraglutide are approved for obesity management in those 12 and older. With recent prescribing patterns showing a predilection for liraglutide, including off‐label use prior to its approval for use in paediatric T2DM [9]. Medications are often used off‐label when standard care methods fail and FDA‐approved medications have been exhausted [9].

Locally, we have seen an increased burden of T2DM in youth [10], and we aimed to evaluate the effectiveness of both on‐ and off‐label GLP‐1 receptor agonists in paediatric patients with T2DM in a real‐world setting. Unlike clinical trials, we examine the real‐world outcomes of GLP‐1 receptor agonists over a 2‐year period. As novel agents become more available for on‐label use in the paediatric population, understanding their effectiveness in non‐clinical trial settings is paramount.

## 2 Methods

### 2.1 Data Source

This IRB‐approved protocol consisted of a single‐center retrospective medical record review of paediatric patients with type 2 diabetes mellitus (T2DM) initially prescribed a GLP‐1 receptor agonist from August 2019 to August 2023. Patients were identified using the International Classification of Disease 10 code of T2DM (E11.xx) and an active or historical prescription for any drug in the GLP‐1 receptor agonist class (semaglutide, liraglutide, dulaglutide, exenatide). Inclusion criteria were: T2DM diagnosis, 0 to 18 years of age at start of GLP‐1 receptor agonist or dual agent, and at least one follow‐up appointment within 24 months after starting GLP‐1. Exclusion criteria were: type 1 diabetes or prediabetes diagnosis, age over 18 at start of GLP‐1 receptor agonist, and absence of follow‐up appointment. If conflicting ICD‐10 codes were present (e.g., both E10.65 and E11.65 indicating type 1 diabetes and T2DM or R73.0 and E11.9 indicating pre‐diabetes and T2DM), manual chart review was done to confirm the accuracy of the diagnosis. Patients with positive pancreatic autoantibodies were excluded. In cases of mixed ICD‐10 codes for both prediabetes and T2DM, T2DM was confirmed on manual chart review when patients met the American Diabetes Association's standard diagnostic criteria, (confirmed hyperglycemia > 200 mg/dL in an individual with symptoms of hyperglycemia, or two confirmatory measurements (HbA1c, fasting glucose, or oral glucose tolerance test) of glycemia in the diabetes range) [11].

Data collection occurred at four time points during this period. Visit 1—the baseline visit—was the visit at which a GLP‐1 receptor agonist was first prescribed to the patient. Visit 2 was a 6‐month follow‐up with acceptable visits falling within 4–8 months. Visit 3 was a one‐year follow‐up with acceptable visits falling within 10–14 months. Visit 4 was a two‐year follow‐up with acceptable visits falling within 22–26 months. If no appointments fell within visit timeframes, no data was collected for that follow‐up interval.

At the baseline visit, we also abstracted the following information from medical records: legal sex, birthdate, race, ethnic group, insurance, and T2DM diagnosis date. For each visit, the following information was abstracted from medical records: HbA1c, BMI, weight, metformin use, insulin use, GLP‐1 receptor agonist prescribed, AST, ALT, LDL, HDL, triglyceride, and total cholesterol.

### 2.2 Statistical Analysis

Data analyses were performed using JMP Pro version 16. Normally distributed continuous variables were summarised using means ± standard deviations (SDs). For skewed continuous variables, data were summarised with medians and interquartile ranges (IQRs). The normality of the variables was assessed through histogram evaluation, normal quantile plots, and the Shapiro–Wilk test. For categorical variables, frequencies and percentages were reported. We analysed significant differences between samples with an independent likelihood ratio statistical test or Fisher exact test to determine respective *p* values. All hypothesis tests were two‐tailed, using a *p*‐value of < 0.05 to indicate statistical significance.

## 3 Results

### 3.1 Subjects

A total of 340 patients met inclusion criteria. Nine were lost to follow‐up, nine discontinued medication due to adverse effects, and one discontinued medication due to cost. A total of 321 patients were included in the analysis. The average age at GLP‐1 receptor agonist start date was 14.5 ± 2.5 years. A majority (*n* = 194, 60.4%) of patients were female. Most patients (*n* = 213, 66.4%) self‐identified as non‐Hispanic Black, followed by non‐Hispanic white (*n* = 57, 17.8%) and Hispanic (*n* = 44, 13.7%). The remainder identified as Other (*n* = 4, 1.2%), Asian‐Pacific Islander (*n* = 2, 0.6%), or declined to answer (*n* = 1, 0.3%). A significant majority of patients were on Medicaid (*n* = 230, 71.7%). Two patients (0.6%) had no insurance, and the remainder (*n* = 89, 27.7%) had private insurance.

### 3.2 Retention and Clinical Data Available

Per inclusion criteria, all patients had at least one follow‐up visit. Three hundred twenty‐one patients had a baseline visit, 222 had a 6‐month, 174 had a 12‐month, and 89 had a 24‐month follow‐up visit. As this was a retrospective review rather than a prospective research protocol, not all patients had all data of interest available for each visit. Clinical data was obtained at the discretion of the treating endocrinologist, and available data was used for analysis.

### 3.3 Effect of GLP‐1 Receptor Agonist Therapy on HbA1c

At baseline, mean HbA1c was 8.3% ± 2.5%. After using GLP‐1 agonist for 6 months, the mean HbA1c was 7.2% ± 2.2%. At 12 months, mean HbA1c was 7.4% ± 2.3% (*p* < 0.001). At 24 months, mean HbA1c was 7.9% ± 2.5% (*p* = 0.24). See Table 1.

**TABLE 1:** Baseline and follow‐up metabolic variables.

	Baseline (*n* = 321)	6‐month (*n* = 222)	12‐month (*n* = 174)	Baseline to 12‐month	24‐month (*n* = 89)	Baseline to 24‐month
HbA1c	8.3 ± 2.5	7.2 ± 2.2	7.4 ± 2.3	**< 0.001**	7.9 ± 2.5	0.24
	(*n* = 312)	(*n* = 219)	(*n* = 169)		(*n* = 85)	
Total cholesterol	171.4 ± 42.6	164.0 ± 38.5	163.5 ± 38.0	0.17	166.6 ± 37.8	0.73
	(*n* = 140)	(*n* = 82)	(*n* = 95)		(*n* = 50)	
Triglycerides	184.2 ± 180.2	146.9 ± 83.1	149.9 ± 128.00	0.21	140.9 ± 105.4	0.69
	(*n* = 140)	(*n* = 80)	(*n* = 95)		(*n* = 50)	
LDL	115.2 ± 38.0	113.6 ± 38.8	110.0 ± 38.8	0.53	115.2 ± 41.4	0.75
	(*n* = 140)	(*n* = 83)	(*n* = 97)		(*n* = 50)	
HDL	39.5 ± 8.5	38.3 ± 8.0	39.1 ± 9.2	0.65	39.6 ± 9.2	0.42
	(*n* = 140)	(*n* = 83)	(*n* = 96)		(*n* = 50)	
ALT	36.8 ± 40.3	33.4 ± 38.3	34.5 ± 43.5	0.61	34.6 ± 44.7	0.17
	(*n* = 208)	(*n* = 166)	(*n* = 143)		(*n* = 72)	
AST	25.3 ± 20.0	24.8 ± 26.2	24.5 ± 21.8	0.38	25.5 ± 29.2	0.99
	(*n* = 208)	(*n* = 165)	(*n* = 143)		(*n* = 72)	
BMI	38.0 ± 7.4	37.4 ± 7.3	38.1 ± 7.0	0.58	38.4 ± 7.9	0.35
	(*n* = 317)	(*n* = 217)	(*n* = 172)		(*n* = 89)	
Weight	103.9	104.5	106.2	**0.003**	105.4	**0.002**
	(*n* = 317)	(*n* = 219)	(*n* = 172)		(*n* = 89)	

*Note:* The bolded values are the statistically significant *p*‐values (< 0.05) for baseline to 12 month comparison and baseline to 24 month comparisons.

Abbreviations: ALT, alanine aminotransferase; AST, aspartate aminotransferase; BMI, body mass index; HbA1c, haemoglobin A1c; HDL, high‐density lipoprotein; LDL, low‐density lipoprotein.

### 3.4 Differences in Pharmacological Management From Baseline

**FIGURE 1:**
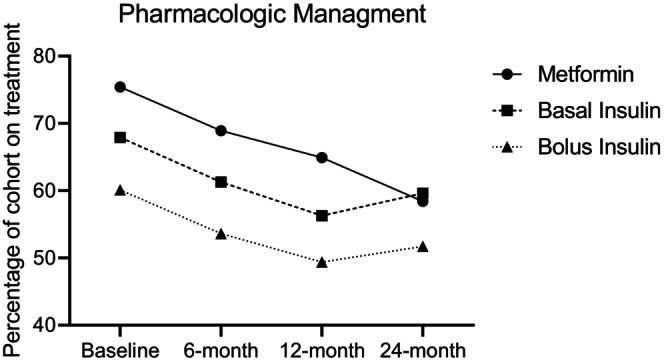
Changes in pharmacological management of T2DM for patients on GLP‐1 receptor agonist treatment over a two‐year period.

At baseline, 75.4% (*n* = 242) of patients were also taking metformin, 67.9% (*n* = 218) required basal insulin, and 60.1% (*n* = 193) were on bolus insulin. The proportion of patients on metformin at 6 months was 68.9% (*p =* 0.10), 12 months was 64.9% (*p* = 0.014), and 24 months was 58.4% (*p* = 0.002). The proportion of patients on basal insulin at 6 months was 67.9% (*p =* 0.11), 12 months was 61.3% (*p* = 0.010), and 24 months was 59.6% (*p =* 0.14). The proportion of patients on bolus insulin at 6 months was 53.6% (*p =* 0.14), 12 months was 49.4% (*p* = 0.022), and 24 months was 51.7% (*p =* 0.15). See Figure 1. The most prescribed GLP‐1 receptor agonist was dulaglutide (73.5%), followed by semaglutide (14.5%), and liraglutide (11.8%). In a total of 805 prescriptions, one was for tirzepatide (0.1%).

### 3.5 Differences in BMI and Other Metabolic Health Parameters

At baseline, mean BMI was 38.0 ± 7.4 kg/m^2^. There was no significant change in BMI; however, there was a change in weight from baseline that persisted at both 12 and 24 months (See Table 1). Regarding other metabolic parameters, including total cholesterol, triglycerides, LDL, HDL, and liver transaminases (ALT, AST), there were no significant changes at either 12 or 24 months (See Table 1).

## 4 Discussion/Conclusion

The burden of T2D in youth is increasing and disproportionally affects minority [10, 12] and economically disadvantaged youth [10]. Health burden is high, with an estimated reduction in life expectancy of 15 years [13]; however, the advent of GLP‐1 receptor agonist therapy shows significant promise. The use of GLP‐1 receptor agonists in youth with T2D who are uncontrolled with metformin is now considered the standard of care [14].

Randomised controlled trials have consistently shown benefit in haemoglobin A1c and other glycemic metrics when used in paediatric patients with T2D [3] with estimated treatment differences of up to 1.4% relative to placebo [15]. These improvements, however, reflect intensive research protocols, with some patients getting 8 contact points with research staff over a 26‐week period [15], a level of supervision and support not feasible in clinical practice. As the use of GLP‐1 receptor agonists in the management of paediatric T2D becomes more routine and standard of care [14], we aimed to add to the existing literature [3, 15, 16] by analysing the effects of GLP‐1 receptor agonists on metabolic health and insulin needs in real‐world settings for a paediatric cohort with T2D in the southeastern United States, an area with a particularly high diabetes burden [17].

In this cohort of patients on GLP‐1 receptor agonist therapy for up to 24 months, we observed an initial decrease in HbA1c at 6 and 12 months in line with prior studies [3] that was not sustained at 24 months. While HbA1c improvements did not persist, the use of additional agents such as insulin and metformin decreased when GLP‐1 receptor agonists were used, suggesting that GLP‐1 receptor agonists may improve glycemic control with less polypharmacy, thereby lowering treatment burden. While metformin is standard of care for both youth and adults with T2D, adherence is problematic, with many patients being unable to consistently take this twice daily medication [18]. Having additional agents available increases options for shared decision making amongst patients, families, and providers, ensuring that medical needs are being addressed while finding treatment options in line with patients' goals and preferences.

At study onset, liraglutide and semaglutide were the only GLP‐1 receptor agonists approved by the FDA for use in paediatric patients with T2D. While dulaglutide was approved for use in paediatric patients with T2DM aged 10 and older on November 17th, 2022, 76.0% of our baseline visits occurred prior to that date. However, most patients in our cohort were prescribed dulaglutide, likely reflecting local insurance coverage (which does not always reflect FDA approval) in addition to patient preference for a weekly rather than a daily option. Off‐label use of GLP‐1 agonists in younger ages was done at the discretion of the treating physician in families who agreed to their use.

While studies using GLP‐1 receptor agonists in the management of paediatric patients with obesity without T2D show promise related to weight management [16, 19, 20, 21], studies in adolescents with T2D do not show consistent effects on BMI [15, 22, 23]. Our study adds to this body of data, showing minimal effect of GLP‐1 receptor agonists on BMI in youth with T2D. While the lack of improvement in BMI is disheartening, the lack of significant weight gain should be noted, particularly as insulin, the mainstay in paediatric T2D management prior to GLP‐1 receptor agonists, is associated with weight gain. Similar to weight, we found no significant change—either improvement or worsening—in baseline and 12‐ or 24‐month metabolic markers including cholesterol, triglycerides, or liver enzymes.

## 5 Limitations

When analysing data in a real‐world setting, there are certain limitations to be expected. The greatest limitation in a study investigating the effectiveness of pharmaceutical intervention is medication adherence. Patients with improper medication adherence, whether intentional or unintentional, may experience different outcomes compared to those who comply fully. Patients who were prescribed a GLP‐1 receptor agonist but reported not taking the medication were excluded from our analysis. The reason for cessation was not consistently documented and was therefore unable to be evaluated in this analysis. Additionally, the presence or absence of hypoglycemia was not consistently documented in clinical data and therefore we are unable to comment on the rate of this side effect in patients in this cohort.

Additionally, not all patients had a full 2 years of follow‐up data, which may have contributed to the insignificant decrease in HbA1c at the 2‐year follow‐up relative to the significant decrease observed at the 1‐year follow‐up. The lack of 2‐year follow‐up data was related to several factors. First, patients with baseline visits in 2023 had not had sufficient time for 2 years of treatment at the time our data was collected. Additionally, some patients had visits occur outside of the specified visit range described in the Section 2. Finally, some patients were lost to follow‐up or transitioned to adult care.

Finally, significant individual variation was appreciated. While the mean HbA1c change was modest, some patients had a significant reduction in HbA1c while others showed minimal improvement. This could be due to a true lack of biological response to GLP‐1 receptor agonists in certain individuals, difficulty taking the prescribed medication, or additional factors such as dietary and activity habits. Further work is needed to better predict which patients will benefit most from available therapeutic options.

Long‐term follow‐up studies are needed to better understand the weight and metabolic effects of GLP‐1 receptor agonists, as well as the reduction in insulin requirements in the management of paediatric T2D. In addition to observing the effects on metabolic health, data collection can also be used to compare GLP‐1 receptor agonists based on efficacy in real‐world settings, adherence, adverse effects, and accessibility.

## Author Contributions

A.G.B. helped acquire the data, assisted in performing data analysis, authored the first draft of the manuscript, and edited subsequent drafts. C.A.G.‐V. performed data analysis, contributed to manuscript writing, and edited drafts. J.A.S. conceptualised and designed the study, assisted with acquiring the data, reviewed the data analysis, edited manuscript drafts, contributed to manuscript writing, and approved the final manuscript.

## Disclosure

J.A.S. has nothing to disclose. C.A.G.‐V. has nothing to disclose. A.G.B. is supported by the National Center for Advancing Translational Sciences of the National Institutes of Health under award number TL1TR003106. Research reported in this publication was supported by the National Center for Advancing Translational Sciences of the National Institutes of Health under award number TL1TR003106. The content is solely the responsibility of the authors and does not necessarily represent the official views of the National Institutes of Health.

## Ethics Statement

This study was approved by the University of Alabama at Birmingham (UAB) Institutional Review Board (IRB), approval number IRB‐300008263.

## Conflicts of Interest

The authors declare no conflicts of interest.

## Data Availability

Data are available upon request from the corresponding author with approval from the University of Alabama at Birmingham Institutional Review Board.
